# Enhancing Airport Traffic Flow: Intelligent System Based on VLC, Rerouting Techniques, and Adaptive Reward Learning †

**DOI:** 10.3390/s25092842

**Published:** 2025-04-30

**Authors:** Manuela Vieira, Manuel Augusto Vieira, Gonçalo Galvão, Paula Louro, Alessandro Fantoni, Pedro Vieira, Mário Véstias

**Affiliations:** 1 Electronics Telecommunication and Computer Department, Instituto Superior de Engenharia de Lisboa-Instituto Politécnico de Lisboa, 1949-014 Lisboa, Portugal; manuela.vieira@isel.pt (M.A.V.); a45903@alunos.isel.pt (G.G.); paula.louro@isel.pt (P.L.); mario.vestias@isel.pt (M.V.); 2 UNINOVA-CTS and LASI, Quinta da Torre, Monte da Caparica, 2829-516 Caparica, Portugal; 3 DEE NOVA School of Science and Technology, Quinta da Torre, Monte da Caparica, 2829-516 Caparica, Portugal; 4 Instituto de Telecomunicações, Instituto Superior Técnico, 1049-001 Lisboa, Portugal; 5 INESC-INOV, Instituto Superior Técnico, Universidade de Lisboa, 1000-029 Lisboa, Portugal

**Keywords:** visible light communication (VLC), deep reinforcement learning (DRL), indoor localization, autonomous guided vehicles (AGVs), adaptive reward mechanisms, multi-agent systems, route optimization, wayfinding assistance, traffic flow simulation, intelligent rerouting techniques

## Abstract

**Highlights:**

**What are the main findings?**

- The AI and VLC-based airport traffic management system enhances traffic flow efficiency, reduces congestion, and enhances safety through real-time asset tracking, adaptive reward mechanisms, and intelligent rerouting strategies.
- VLC-enabled infrastructure: Utilizes tetrachromatic LED luminaires and SiC optical receivers in a hybrid mesh network to provide precise, location-specific guidance and replace traditional gateway systems.

**What is the implication of the main finding?**

- AI-driven optimization: Employs Deep Reinforcement Learning (DRL) with Q-learning to support adaptive reward allocation, dynamic phase control, and intelligent rerouting across multiple intersections.
- GPS-free indoor localization: Enables seamless navigation for AGVs and pedestrians through VLC-based geolocation, contributing to smoother operations, improved mobility, and an enhanced passenger experience.

**Abstract:**

Airports are complex environments where efficient localization and intelligent traffic management are essential for ensuring smooth navigation and operational efficiency for both pedestrians and Autonomous Guided Vehicles (AGVs). This study presents an Artificial Intelligence (AI)-driven airport traffic management system that integrates Visible Light Communication (VLC), rerouting techniques, and adaptive reward mechanisms to optimize traffic flow, reduce congestion, and enhance safety. VLC-enabled luminaires serve as transmission points for location-specific guidance, forming a hybrid mesh network based on tetrachromatic LEDs with On-Off Keying (OOK) modulation and SiC optical receivers. AI agents, driven by Deep Reinforcement Learning (DRL), continuously analyze traffic conditions, apply adaptive rewards to improve decision-making, and dynamically reroute agents to balance traffic loads and avoid bottlenecks. Traffic states are encoded and processed through Q-learning algorithms, enabling intelligent phase activation and responsive control strategies. Simulation results confirm that the proposed system enables more balanced green time allocation, with reductions of up to 43% in vehicle-prioritized phases (e.g., Phase 1 at C1) to accommodate pedestrian flows. These adjustments lead to improved route planning, reduced halting times, and enhanced coordination between AGVs and pedestrian traffic across multiple intersections. Additionally, traffic flow responsiveness is preserved, with critical clearance phases maintaining stability or showing slight increases despite pedestrian prioritization. Simulation results confirm improved route planning, reduced halting times, and enhanced coordination between AGVs and pedestrian flows. The system also enables accurate indoor localization without relying on a Global Positioning System (GPS), supporting seamless movement and operational optimization. By combining VLC, adaptive AI models, and rerouting strategies, the proposed approach contributes to safer, more efficient, and human-centered airport mobility.

## 1. Introduction

Navigating crowded indoor environments like shopping malls, airports, and hospitals is often hindered by confusing layouts, crowded pathways, and the limitations of traditional navigation tools such as GPS. These challenges can lead to frustration, stress, and safety concerns, particularly for individuals unfamiliar with the environment or those with visual impairments. Efficient indoor navigation is essential to improving accessibility, enhancing user experiences, and ensuring safe and smooth movement in complex spaces.

Current indoor positioning methods mainly rely on technologies such as Wi-Fi, Bluetooth, Radio-Frequency Identification (RFID), Visible Light Communication (VLC), and inertial navigation [1,2,3,4,5]. Although a variety of techniques are available—including Wi-Fi-based approaches [6,7], and visual indoor topology localization methods [8,9]—many of these require dense deployment of Wi-Fi access points or costly sensors to achieve accurate positioning. Recent studies using VLC have shown promising results in indoor localization tasks. For instance, Oh and Kim [10] employed Double Deep Q-Networks (DDQNs) in VLC-enabled offices, and Affan et al. [11] applied machine learning to overcome signal shadowing in VLC-based P-NOMA systems. Abdalmajeed et al. [12] enhanced localization precision using advanced optical signal modeling and learning-based decoding. However, these efforts focus primarily on user localization, signal processing, or channel modeling in simplified or static indoor environments. They typically lack consideration for real-time coordination of multiple mobile entities, particularly in high-traffic environments like airports. Visible Light Communication (VLC) [13,14,15] offers a promising alternative by leveraging existing LED lighting infrastructure to enable both illumination and data transmission.

VLC relies on low-cost white LEDs that emit modulated visible light, imperceptible to the human eye, which is detected and decoded by mobile receivers equipped with photodetectors [16,17,18]. Advanced techniques such as Wavelength Division Multiplexing (WDM) further enhance VLC’s data rates, enabling precise indoor localization. By dividing indoor spaces into spatial light beams identified by unique signal sequences, VLC systems can deliver fine-grained positioning accuracy. Additionally, the use of multicolored LED luminaires introduces enhanced possibilities for signal modulation and detection within VLC systems [19]. In particular, white polychromatic LEDs enable Wavelength Division Multiplexing (WDM), a technique that significantly boosts data transmission capacity. In this work, a WDM receiver based on an amorphous SiC double PIN/PIN heterostructure was employed, functioning as a light-controlled optical filter [20]. This receiver allows the simultaneous reception of multiple optical channels, which are subsequently processed through amplification, switching, and wavelength conversion stages, before the transmitted signals are decoded to retrieve the original information. This approach considerably improves indoor navigation performance by delivering high-precision localization, thereby enriching the user experience in densely populated indoor environments [9,21,22].

Airports are dynamic environments requiring efficient navigation for pedestrians and Autonomous Guided Vehicles (AGVs). This study integrates Artificial Intelligence (AI) and Visible Light Communication (VLC) technologies to optimize traffic flow, reduce congestion, and enhance safety. AI agents equipped with VLC transmitters and receivers track and manage assets in real time, while VLC-enabled luminaires provide location-specific guidance through modulated light signals.

Deep Reinforcement Learning (DRL) is incorporated to dynamically adapt positioning and routing strategies based on real-time environmental conditions. DRL models learn from user interactions, obstacles, and contextual changes, enabling seamless navigation in crowded, unpredictable spaces [23,24,25]. This adaptability supports precise guidance to gates, check-ins, and amenities, improving user experiences and operational efficiency.

In addition, this work introduces an adaptive reward mechanism [26,27,28,29] designed to fine-tune agent behavior by integrating both horizontal and vertical shared artery management and pedestrian flow prioritization. This mechanism enhances the learning process by balancing the needs of vehicle storage, congestion relief, and pedestrian safety across interconnected traffic arteries.

For airport operators, the proposed system ensures efficient asset tracking, streamlines operations, and supports real-time decision-making. The VLC-DRL integration, reinforced by adaptive reward modeling, improves scalability and performance, offering personalized services and intelligent coordination in complex indoor environments.

Building on this communication framework, we propose a Deep Reinforcement Learning (DRL)-based traffic management system for dynamic indoor environments, specifically airport terminals. The system integrates VLC-enabled luminaires and AI agents equipped with VLC transmitters/receivers to manage both pedestrian and AGV flows. Unlike traditional models, this framework considers a multi-agent context and adapts to real-time environmental conditions. Furthermore, an adaptive reward mechanism is introduced to guide the learning process by incorporating metrics for pedestrian safety, AGV storage optimization, and congestion mitigation in both vertical and horizontal indoor arteries. This mechanism allows the DRL model to learn optimal phase policies that may exclusively favor pedestrians or AGVs when necessary, enhancing operational safety and efficiency. By unifying VLC and DRL technologies, and addressing both navigation and traffic management challenges in crowded environments, this work contributes a scalable and intelligent framework tailored for modern airport infrastructures.

To the best of our knowledge, no existing studies address the joint management of pedestrian and baggage flows within airport terminals using an intelligent traffic coordination framework. Even fewer have explored the use of Visible Light Communication (VLC) for real-time navigation and multi-agent coordination in such complex indoor environments. This work proposes a novel architecture that integrates VLC-based geolocation with adaptive Deep Reinforcement Learning (DRL) for dynamic traffic control of Automated Guided Vehicles (AGVs), aiming to improve fluidity, coordination, and safety across multiple intersections within airports.

The paper is structured as follows: Section 1 introduces the motivation and key contributions. The remainder is structured as follows. Section 2 presents the related work, reviewing current research on AI-based traffic control systems, VLC-enabled communication infrastructures, rerouting techniques, and pedestrian integration strategies in high-density environments. Section 3 describes the proposed AI and VLC-integrated traffic management system, including the network topology, communication protocols, and the role of VLC-enabled luminaires in indoor localization. Section 4 details the AI optimization model and rerouting strategy, focusing on the implementation of DRL and Q-learning for intelligent decision-making, adaptive rerouting, and traffic phase control. Section 5 introduces the adaptive reward mechanism with horizontal and vertical shared arteries and pedestrian integration, presenting the design and evaluation of the reward structure and highlighting its impact on pedestrian prioritization, traffic distribution, and overall system responsiveness through simulation results. Finally, Section 6 presents the conclusions, summarizing the main findings and emphasizing the benefits of integrating VLC and AI with adaptive rewards and rerouting techniques for efficient and safe airport traffic management, while also outlining future research directions.

## 2. System Design and Methodological Approach

The objective is to design a VLC-based guidance system and define use cases for mobile users in large multi-terminal airports.

### 2.1. Fundamentals of Visible Light Communication (VLC)

**Figure 1:**
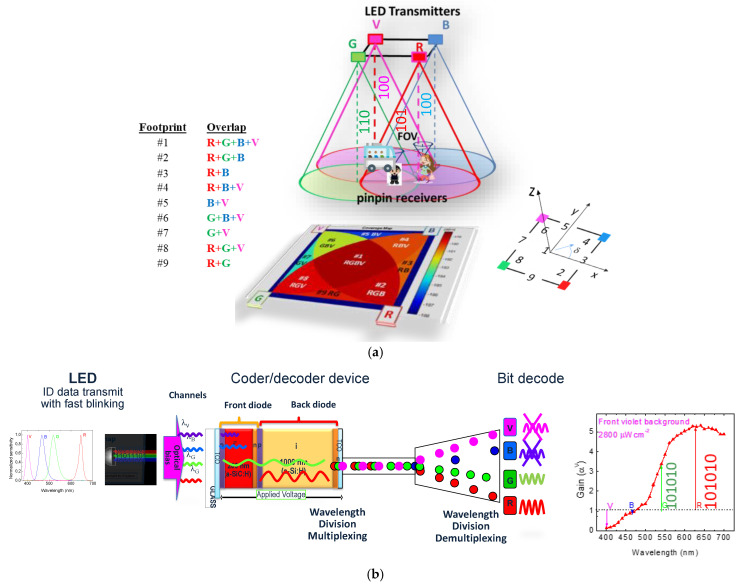
(**a**) 3D spatial arrangement of transmitters and receivers, including their footprints and coverage map within the square topology. (**b**) Structure and operating principle of the PIN/PIN receiver, along with the spectral gain under violet front optical bias (αV). The arrows indicate the optical gain corresponding to the analyzed R, G, B, and V input channels.

The system comprises two main modules, transmitter and receiver, as shown in Figure 1a. For communication and lighting, white light tetra-chromatic LEDs (WLEDs) are used, with distinct data channels for each chip. Each luminaire comprises four WLEDs framed at the corners of a square unit cell. Only one chip per node transmits data, using specific wavelengths and power densities: red (626 nm, 25 μW/cm^2^), green (530 nm, 46 μW/cm^2^), blue (470 nm, 60 μW/cm^2^), and violet (400 nm, 150 μW/cm^2^). The remaining LEDs maintain white light perception, and luminous intensity is regulated via driving currents [9]. The transmitter converts data into bytes and then into light signals using the OOK modulation. This method uses the presence or absence of light for data transmission, allowing for adaptive data rates that optimize performance under varying conditions. The OOK modulation scheme encodes binary data (“1” and “0”) through light pulses, making it efficient, robust, and cost-effective while seamlessly integrating with LED lighting systems. Additionally, OOK simplifies signal processing and enhances reliability through its use of light pulses. Once transmitted via the optical channel, the modulated light is received by a VLC receiver that processes and decodes the transmitted data. This system achieves reliable, efficient data transmission while providing illumination in large indoor environments.

The receiver, illustrated in Figure 1b, utilizes a multiplexer (MUX) photodetector with a p-i′(a-SiC:H)-n/p-i(a-Si:H)-n heterostructure, functioning as an active optical filter. Its design incorporates an intrinsic a-SiC layer optimized for blue light and an a-Si layer for green and red light, enabling wavelength-specific signal processing. These layers filter and detect light signals, converting them into electrical signals that are subsequently decoded to extract data. Using Plasma Enhanced Chemical Vapor Deposition (PECVD), a double-PIN heterostructure is deposited on transparent glass substrates to form the core of the optoelectronic sensor. It features low-doped, high-resistivity layers, providing excellent photosensitivity and conductivity under visible and near-infrared light. Transparent ITO contacts on both sides facilitate light transmission, while back contacts define the active sensor area, ensuring precise detection and enhanced performance [17].

The working principle combines pulsed communication channels of red, green, blue, and violet light (λ_R_, λ_G_, λ_B_, λ_V_), each carrying a specific bit sequence. These channels are absorbed based on their respective wavelengths, with the resulting multiplexed signal analyzed via photocurrent measurement under a negative voltage (−8 V) and 390 nm background lighting. Figure 1b also illustrates the spectral gain, showing wavelength-dependent photocurrent variations. Long wavelengths are amplified, while short wavelengths are suppressed, transforming the device into an active filter. Gain exceeds unity above 500 nm, boosting green and red channels, while violet and blue are attenuated. The voltage is processed through adaptive bandpass filtering, amplification, triggering, and demultiplexing. These steps reconstruct the data signal, involving digital conversion, decoding, and decision-making. This highlights the non-linear characteristics critical for decoding multiplexed signals at the receiver [9,17].

### 2.2. VLC-Based Indoor Positioning

The proposed indoor positioning system leverages Visible Light Communication (VLC) to enable accurate localization within GPS-denied environments, such as airport terminals. The architecture combines triangulation and fingerprinting techniques in a hybrid approach, enhancing both positioning accuracy and system robustness. VLC transmitters, implemented through modulated LED luminaires, emit unique identification codes. The receiver (e.g., embedded in autonomous vehicles or handheld devices) must align within the overlapping zones of multiple transmitters to receive a multiplexed (MUX) signal.

To avoid ambiguity and ensure spatial discrimination, the coverage area is divided into a grid of unit cells, each containing nine distinct fingerprint regions (#1–#9), calibrated to prevent adjacent overlap at the receiver. These fingerprints are defined by the intersection patterns of light signals and the steering angles (δ) of the transmitters. Figure 1a illustrates these overlaps, reference points, and orientations (steering angles δ), which determine the centroid of received coordinates for precise localization. Upon reception, the MUX signal is decoded, and the centroid of the activated regions is computed, providing an accurate estimate of the receiver’s position. Finally, the decoded message is delivered to the user.

This dual-use system capitalizes on existing lighting infrastructure, serving both illumination and communication purposes, which significantly reduces deployment costs and energy consumption. The deterministic propagation of light enables sub-meter positioning accuracy (~20–30 cm) under line-of-sight conditions, outperforming traditional RF-based solutions (e.g., Wi-Fi, Bluetooth), which are typically limited by interference, reflection, and multipath effects. The system is also immune to electromagnetic interference, making it suitable for use in safety-critical environments such as airport runways, hangars, and terminal interiors.

### 2.3. System Architecture and Components

In VLC tracking, geographic coordinates are used to guide users through unfamiliar buildings and help them reach their destinations. This is achieved by dividing the space into cells for positioning, with a Central Manager (CM) overseeing the process and generating optimal routes. The model is based on the building’s layout and the placement of available luminaires, which are displayed on the user’s receiver for orientation. Users can request the CM to guide them to their destination and receive change notifications.

**Figure 2:**
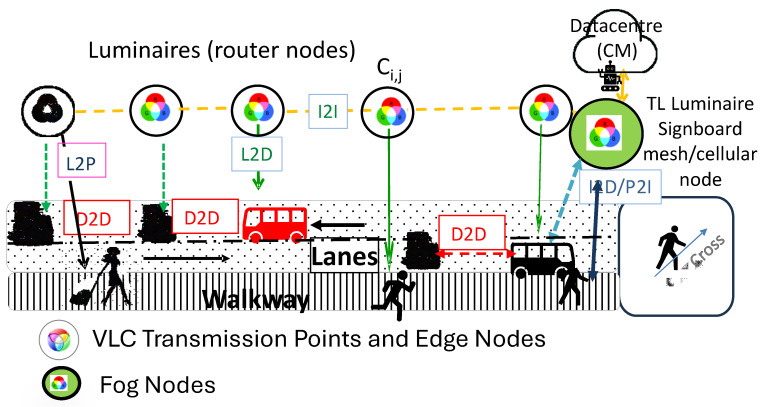
Draft design of a single-lane configuration in the Edge/Frog hybrid architecture.

Introducing this new concept, we present a mesh cellular hybrid structure that offers an innovative approach to network architecture, as shown in Figure 2. The mesh network enables secure, peer-to-peer communication, facilitating direct data exchange among devices (D2D; Device-to-Device). Each WLED emits a unique VLC signal, serving as an identification beacon (L2D; Luminaire-to-Device). This beacon allows the optical receiver to determine the user’s trajectory using a specialized positioning algorithm.

The calculated indoor route, denoted as q(x, y, z, δ, t), encapsulates essential spatial and temporal information, offering users valuable insights into their movement patterns within indoor environments. For example, when a user, either a pedestrian (P) or an AGV, (D), moves between areas and requires navigation assistance (P2I or D2I), they can customize their points of interest to enhance wayfinding services. Each user is assigned an average speed by the Central Manager (CM), which varies according to the type of entity (AGV or passenger). The requested navigation data are then transmitted to the corresponding receiver (I2D/P) through emitters embedded in the infrastructure, such as light traffic controllers or digital signboards [30].

## 3. VLC-Airport Model

The capacity of an airport is strongly influenced by its gateways, boarding areas, and the design of aircraft door zones. Assigning these spaces requires a coordinated approach, customized to meet specific objectives and criteria for each situation. These objectives typically aim to enhance customer service by reducing the travel distances for pedestrians or passengers with luggage, whether they are arriving, in transit, collecting baggage, transferring between terminals, or engaging in shopping activities.

### 3.1. Airport Infrastructure Modeling

Airports are assessed by criteria such as walking distances between facilities and access to ground transportation or parking. Airport capacity relies on the strategic arrangement of gateways, boarding areas, and aircraft door zones. These allocations are designed to meet specific goals, such as reducing travel distances for pedestrians and carriers during key activities like transit, baggage retrieval, and terminal navigation.

While pedestrian walking speeds within airport terminals are not well-documented, studies on pedestrian behavior in similar environments provide insights [31,32]. Pedestrians generally slow down at decision points, such as corridor junctions, terminal entrances, concession areas, and destinations like gates and baggage claim zones [33,34]. Observations show no significant differences in average walking speeds between airport pedestrians and those in other transportation facilities. However, some pedestrians reduce their walking speeds near areas influenced by information systems or environmental cues.

Given the potential for high-density crowd scenarios, especially during peak hours or delayed flight events, there is an increasing need to prioritize pedestrian movement within airport environments. Large gatherings of people can significantly impact flow efficiency and pose safety risks if not properly managed. The proposed system addresses this by integrating adaptive reward mechanisms that dynamically adjust traffic prioritization based on congestion levels and pedestrian density. This ensures timely intervention in congested zones, allowing the system to favor pedestrian phases when clusters are detected, thus enhancing both safety and navigability in critical terminal areas. As highlighted by recent research, crowd-aware traffic systems must proactively respond to pedestrian aggregation to prevent bottlenecks and safety hazards in smart infrastructures [35].

**Figure 3:**
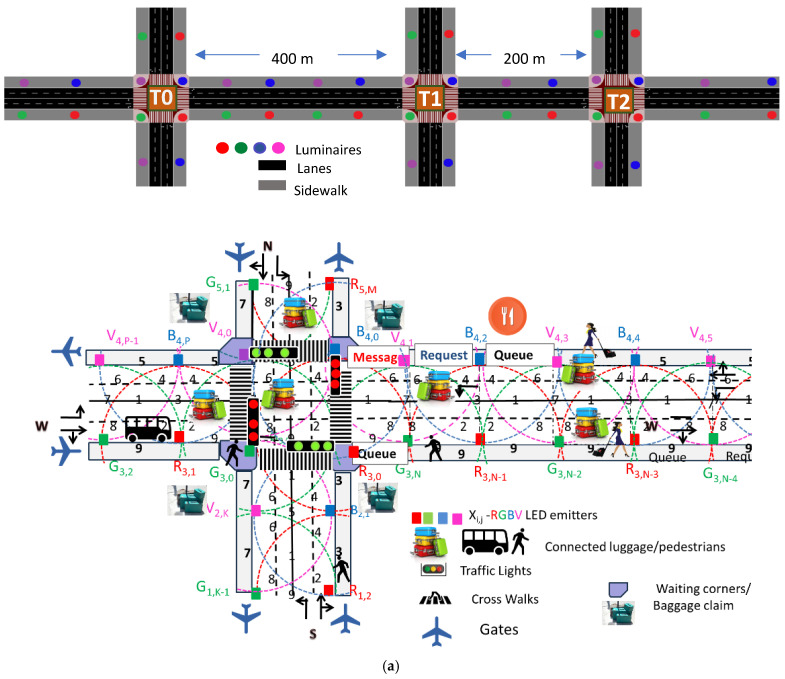

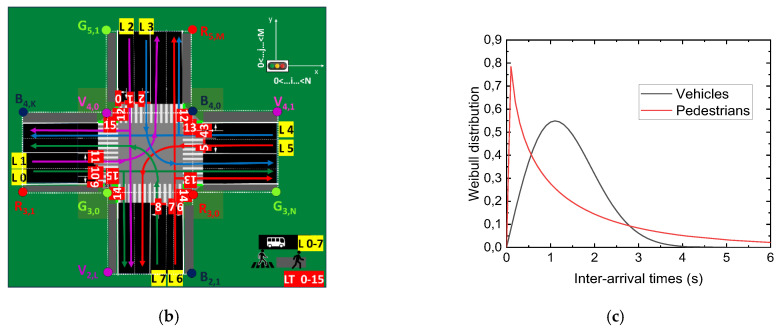
(**a**) Simulated scenario and environment, showing the optical infrastructure (Xij), generated footprints (1–9), and the flow of connected luggage and pedestrians. (**b**) Schematic diagram of Terminal 2, with coded lanes (L/0–7), traffic lights (TL/0–15) and traffic flow (color arrows). (**c**) Weibull distribution.

This study enhances the understanding of pedestrian behavior within terminal corridors, offering valuable insights for optimizing terminal layouts and dynamic routing strategies. Figure 3 presents the simulated airport environment, showcasing the optical infrastructure (Xij), generated footprints, vehicles, and pedestrian flow patterns, and a schematic of the terminal layout. The proposed model leverages multi-level airport footprints and positional data from RGBV luminaires (Xi,j), which are displayed on user devices to facilitate navigation (see Figure 2).

#### 3.1.1. Simulated Airport Scenario

The simulation (Figure 3a) models only one horizontal arterial with three terminals, Terminal 0 for international flights and Terminals 1 and 2 for domestic flights, each with four 4-way arms (N, S, E, W). Arms include two moving lanes (L 0–7) and two sidewalks. Terminals are spaced at 400 m and 200 m intervals. Interiors feature shopping, dining, and rest areas. Figure 3b highlights Terminal 2’s layout, coded lanes (L 0–7), and traffic lights (TL 0–15).

#### 3.1.2. Traffic Management System

Traffic flows along four cardinal points, using binary request-and-response segments for decisions (e.g., turn left/straight or right). Passenger lanes, waiting zones, and crosswalks are integrated. Right lanes handle right turns and straight routes, while left lanes serve left turns. Centralized traffic lights (TL 0–15) are controlled by a Central Manager (CM) to prevent collisions (Figure 3c).

#### 3.1.3. Pedestrian Navigation

Pedestrians move freely on sidewalks in both directions. Destination targeting requires user requests to the CM (D/P2I), with notifications for floor changes. Routes are transmitted via response messages (I2D/P) through traffic signals, acting as routers or mesh nodes (Figure 2).

This request/response framework delivers landmark-based instructions, directing carriers at key decision points and verifying the correct routes. The system improves accessibility, navigation, and safety in airport terminals.

#### 3.1.4. Traffic Generation

The Weibull distribution is widely used in traffic generation to model time intervals between events, such as vehicle and pedestrian arrivals at intersections. It effectively captures realistic traffic patterns by adjusting its shape and scale parameters, providing flexibility to represent diverse traffic behaviors.

The probability density function of the Weibull distribution is given by

$$W\left(t,\;k,\lambda \right)=\left(\frac{k}{\lambda }\right)*{\left(\frac{t}{\lambda }\right)}^{k-1}*e^{{-}^{{\left(\frac{t}{\lambda }\right)}^{k}}}$$

where

- k > 0 is the shape parameter, determining the form of the distribution.
- λ > 0 is the scale parameter, representing the characteristic time scale or mean time.
- t denotes the inter-arrival time between events.

Figure 3c illustrates the Weibull distributions for vehicles and pedestrians.

- Vehicles (k = 2, λ = 1.8)—A moderately skewed distribution, capturing clustered arrivals typically observed in traffic flows.
- Pedestrians (k = 0.8, λ = 1.5)—A more spread-out distribution, reflecting higher variability in pedestrian arrivals.

This approach highlights the adaptability of the Weibull distribution for modeling traffic dynamics, enabling realistic simulations of varying arrival patterns.

Given the clustered arrival pattern of vehicles and the more dispersed arrival pattern of pedestrians, traffic control must exists for ensuring both vehicle and pedestrian safety at each stop sign location. Vehicles need to be controlled more rigorous or carefully regulated to avoid congestion or accidents due to rapid and frequent arrivals. For pedestrians, while less frequent arrivals are expected, exclusive pedestrian phases enhance safety by managing potential conflicts with vehicle traffic.

**Figure 4:**
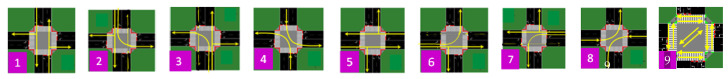
Schematic representation of a single cycle phase diagram, featuring eight vehicular phases (1–8) and a dedicated pedestrian phase (9). Schematic representation of a single cycle phase diagram, featuring eight vehicular phases (1–8) and a dedicated pedestrian phase (9), the arrows are the traffic directions.

Figure 4 visually illustrates the progression of intersection phases (actions) within a structured cycle, consisting of eight AGV phases in the lanes and a dedicated pedestrian phase for walking passengers. Each phase is broken down into discrete time intervals, offering a comprehensive temporal framework [36].

### 3.2. Data Encoding, Decoding Techniques, and Communication Protocol

Data transmission in the airport VLC system follows a synchronous 64-bit data frame structure, utilizing OOK modulation, where binary data are represented by toggling the signal on and off. Each luminaire is equipped with RGBV WLEDs (as shown in Figure 1), enabling the simultaneous transmission of four signals. To accommodate the increased bandwidth, a four-channel filtering receiver is required.

The wavelength-calibrated amplitudes of the RGBV signals produce 16 optical combinations, generating 16 distinct photocurrent levels, which are weighted by optical gain at the photodetector. The PIN/PIN demultiplexer plays a key role in decoding, accurately reconstructing the original message by leveraging calibrated amplitude data. The decoding process relies on the adjustable photon penetration depths into the front and back diodes, as illustrated in Figure 1b. Front irradiation is absorbed early in the front diode, enhancing the electric field in the back diode through the self-bias effect. Red photons penetrate more deeply, improving collection efficiency based on their wavelengths [17].

Each output level is mapped to an n-digit binary code, weighted by optical gain per channel, enabling efficient signal decoding. The system achieves a maximum transmission rate of 100 kbps across four channels. The decoding algorithms are relatively simple, using the background as a selector to choose one of the 2^n^ sublevels (where n represents the number of channels), assigning each sublevel a unique n-bit binary code. The four channels under irradiation represent 2^4^ possible on/off states, with each state encoded as a 4-bit binary [X_1_, X_2_, X_3_, X_4_], where X = 1 if the channel is active and X = 0 if inactive.

To decrypt the received information from the photocurrent signal captured by the photodetector, a crucial step involves retrieving the information through a pre-established calibration curve. This curve, based on the filtering properties of the PIN/PIN photodetector, allows for accurate message decoding. Finally, a parity check is conducted after the word is read to ensure data integrity. Upon decoding the MUX signals and considering the frame structure (Table 1), details such as the pose, transmitter type, and traffic message are revealed.

The communication protocol is designed to ensure synchronization and efficient information exchange, with its structure outlined in Table 1.

The main components of this protocol include the following:

*Frame Structure*
  - *Start of Frame (SoF):* A 5-bit synchronization block ([10101]) marks the beginning of a frame, enabling receivers to align with transmitters.
  - *Identification (ID) Blocks:* Encodes key details using binary representation for decimal numbers, including:
    ○ *Timeline Information:* Contains time data formatted as hour (6 bits), minute (6 bits), and second (6 bits). After this, a flag with the pattern [1111] alerts the decoder to expect specific ID blocks
    ○ *Communication Type (COM):* Specifies the type of exchange, such as Lamp/Infrastructure-to-Device/Pedestrian (L/I 2 D/P), Device-to-Device (D2D), or (D/P 2 I).
    ○ *Transmitter Localization:* Identifies the transmitter’s y, x coordinates.
    ○ *Other Identifiers:* Include additional information specific to the communication type:
      - *Device Number (Device Nr.):* A unique identifier for each device.
      - *Temporary Identification (Device ID):* Session-specific temporary IDs.
      - *Lane Occupancy (Lane 0–7):* Denotes active lane usage.
      - *Requested Traffic Signal (TL 0–15):* Indicates traffic signal requests.
      - *Cardinal Direction (N, S, E, W):* Specifies movement direction.
      - *Active Phase:* Represents the state of “request” or “response” messages at intersections.
*Message Content*
  - *Traffic Message:* The body of the frame includes critical information such as the following:
    ○ *Carrier Information*: Details like Device IDy,x (y,x coordinates), order behind the leader (Nr. behind), and intersection crossing requests.
    ○ *Traffic Payload:* Contains the primary data being transmitted.
*End of Frame (EoF)*
  - A 4-bit block ([0000]) signals the conclusion of the transmission frame, ensuring complete data integrity.

This structured protocol facilitates the seamless encoding and decoding of essential movement data for both vehicles and pedestrians. By maintaining synchronization and ensuring robust data transmission, the system supports efficient and reliable communication within the airport VLC network.

### 3.3. Comparing Framework Algorithms with Empirical Data: Insights from Experimentation

This section describes the algorithms developed to guide users through indoor spaces, including turn-by-turn directions, landmark highlighting, alerts, and alternate route suggestions.

**Figure 5:**
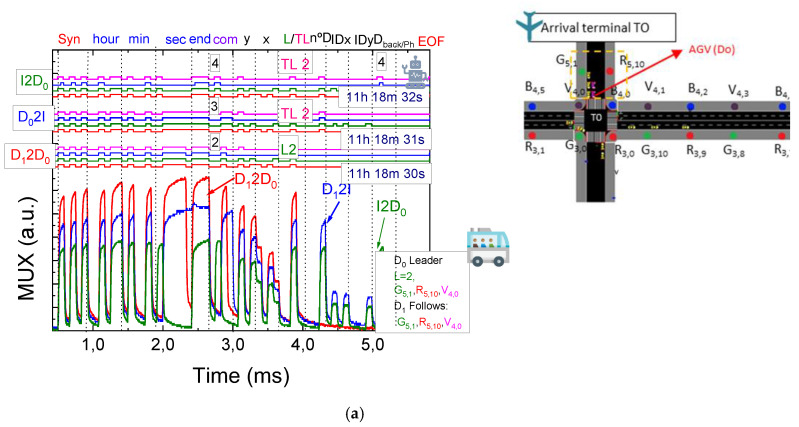

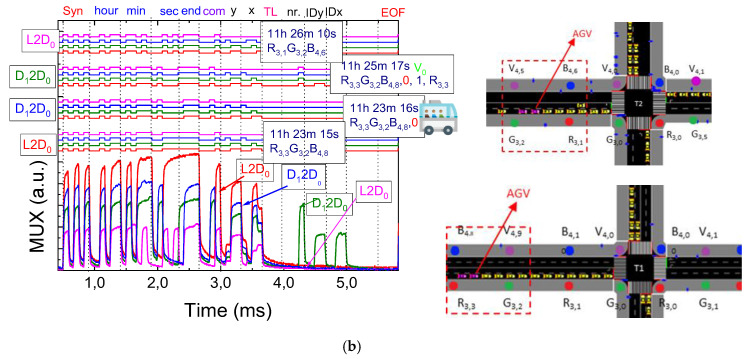
MUX signal distribution across different VLC communication types. The decoded messages are shown at the top, while the analyzed environment is depicted on the right to aid visual interpretation. (**a**) At T0. (**b**) At T1 and T2.

Using Figure 2, Figure 3 and Figure 4, along with the communication protocol detailed in Table 1 and the technique for decoding calibrated signals emitted by transmitters, Figure 5 presents the MUX signals received by the receivers and the decoded optical signals (at the top of the figure). On the right-hand side, the analyzed environment is displayed to assist visual interpretation.

In Figure 5, it is assumed that a flight has just landed at the international terminal T0. The devices, D_0_ and D_1_, originate from this terminal and are en route to domestic terminal T2, the designated location for baggage drop-off. The AGVs stationed at T0, as depicted in Figure 5a, on the right-hand, are awaiting the activation of their phase to execute a left turn. During this interval, various VLC protocols are established and systematically analyzed.

For D2D communication, the rear device (D_1_) communicates with the device ahead (D_0_), transmitting its position (R_5,10_, G_5,1_, V_4,0_), lane number (2), and the number of devices following it (none in this case). The time of the communication (11:18:30) is also included. Since there are no devices behind D_1_, no additional information is passed to the front in these blocks. Upon receiving this communication, the leader (D_0_), which is the first vehicle in the queue, sends a request to the CM via D_0_2I. This request includes the leader’s position (R_5,10_ G_5,1_, V_4,0_), the traffic light (TL) it is referencing (2), the number of devices following it (1), and the time of the communication (11:18:31). The leader also provides the identifier of the device following it (G_5,1_) and indicates that there are no vehicles behind the follower at this moment (0).

Next, an I2D0 communication occurs, where the CM responds with the same information to the leader at 11:18:32, confirming that the active phase is 4 (WE). Once the NS left phase is activated, the vehicles proceed to the central terminal T1, where they queue up, as shown in Figure 5b. At this stage, the devices exchange L2D_0_ and D_1_2D_0_ communications.

In the L2D0 communication, the vehicles are informed about their positions on the lane (R_3,3_, G_3,2_, B_4,8_) along with the time the communication was established (11:23:15). The D_1_2D0 communication, involving the last device in the queue, shares details such as its position (R_3,3_, G_3,2_, B_4,8_), lane (0), the number of devices behind it (currently 0), and the time of the communication (11:23:16). Since no devices follow it, no further information is transmitted. For the next device in the queue, D2D communication is initiated, transmitting the device’s position (R_3,3_, G_3,2_, B_4,8_), lane (0), the number of devices following it (1), the time of the communication (11:23:17), and the position of the device behind it (R_3,3_).

After passing through T1, the devices reach terminal T2, where they queue again, awaiting their phase. At this point, D2D and L2D communications are reestablished. For the L2D0 communication involving the first device under study, for instance, information transmitted includes its position on the lane (R_3,1_, G_3,2_, B_4,6_) and the time the communication is established (11:26:10).

**Figure 6:**
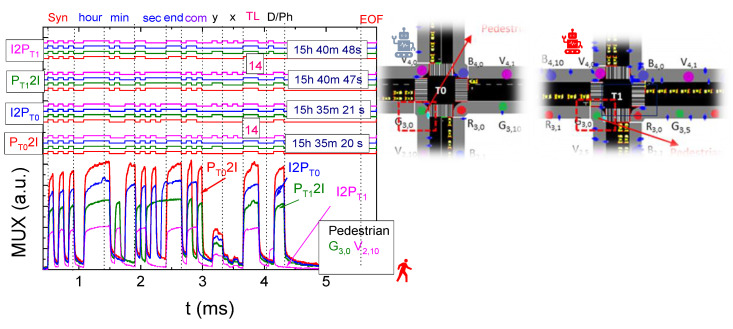
MUX signal allocated to different VLC communication types. On the top the decoded messages are displayed. On the right-hand side, the analyzed environment with the RGBV street lights is displayed to assist visual interpretation.

In Figure 6, it is assumed that a flight has recently arrived at the international terminal T0. Devices D_0_ and D_1_ originate from this terminal and are en route to domestic terminal T2, the designated location for baggage drop-off. The pedestrians positioned at T0, are waiting for their phase to be activated to perform a left turn. During this time, various VLC protocols are initiated and analyzed.

In the context of P2I communication, the pedestrian initially transmits their current position (y, x: G_3,0_ V_2,10_), the corresponding traffic light identifier (TL:14), the intended crossing direction (East, coded as 3), and the timestamp of the communication (15:35:20). In response, I2P message is sent by the Intersection Manager (IM), confirming the pedestrian’s location (G_3,0_ V_2,10_), the associated traffic light (TL:14), the currently active phase (in this case, NS Left, phase 3), and the response timestamp (15:35:21).

After successfully crossing intersection T0, the pedestrian proceeds to a new waiting area at intersection T1, where a similar communication cycle is established. A new P2I message is issued, again including the pedestrian’s position (G_3,0_ V_2,10_), traffic light identifier (TL:14), intended direction (East, 3), and timestamp (15:40:47). The subsequent I2P response from the IM confirms the same position (G_3,0_ V_2,10_), traffic light (TL:14), the currently active phase (now WE, phase 4), and the updated timestamp (15:40:48).

Upon completing the crossing at T1, the pedestrian reaches intersection T2. After a brief interval, the pedestrian may initiate another crossing—either in the same or a different direction (e.g., P_T2_2V). Accordingly, a final exchange of messages occurs: a new P2I message is sent, followed by the corresponding I2P response from the IM.

These communication sequences demonstrate the effectiveness of the VLC system in supporting and detailing the flow of different communication types: V2I, V2V, Pedestrian-to-Infrastructure P2I, and I2P, at multiple intersections. This structured framework enhances the coordination between traffic elements and pedestrian movements, promoting safety and efficiency within the airport environment.

### 3.4. Adaptive Traffic Signal Control Using Deep Reinforcement Learning

**Figure 7:**
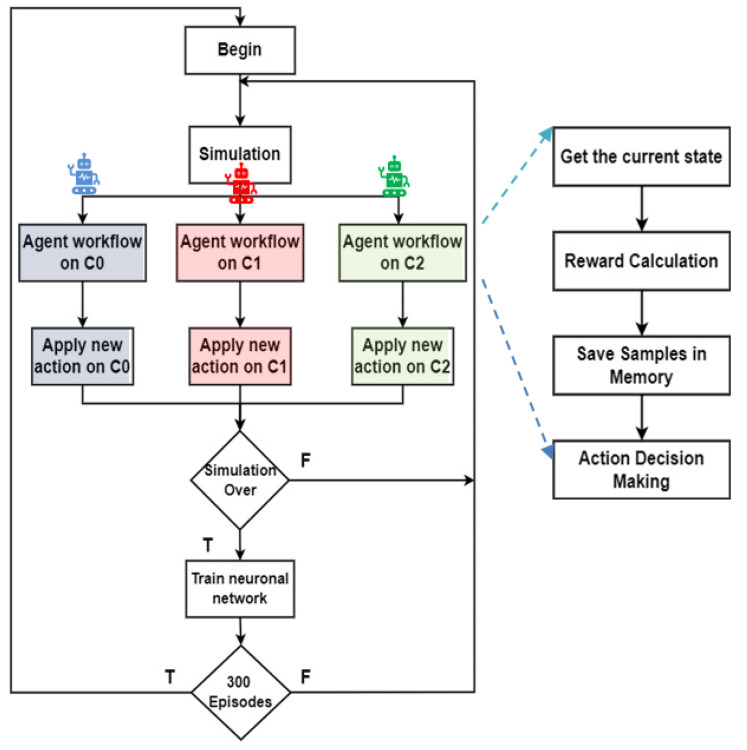
Flowchart during simulation and training.

An intelligent system leveraging data from VLC enables real-time traffic control and response to AGVs and walking passengers’ maneuvers. A Multi-Agent Reinforcement Learning (MARL) system manages three identical four-arm terminals (Figure 3a) with each agent (T0, T1, and T2) mapping its environment into cells to collect vehicle and pedestrian data. Communication between infrastructure and vehicles ensures cooperative information sharing, while shared traffic experiences train a unified network for optimized phase selection. The flowchart during simulation and training is displayed in Figure 7.

A Deep-Q Network (DQN) trained with deep Q-Learning predicts Q-values for actions based on state inputs, addressing traditional Q-Learning’s challenges in large state-action spaces. At each step, *t,* the agent observes the state, *st*, takes an action, *at*, that transforms the state observed to a next state *st* + 1, and calculates a reward *rt*, based on vehicle and pedestrian waiting times.

The system is based on the deep Q-learning (DQL) algorithm, where an agent learns an optimal policy to maximize the cumulative discounted reward over time. The action-value function Q(s,a) is approximated using a deep neural network with parameters θ. The update rule is defined as follows:

(1) $$Q_{target}=r_{t}+\gamma .maxQ_{pred}\left(s_{t+1},a\prime ,\theta -\right)$$

where $r_{t}$ is the reward obtained, γ is a discount factor applied to the ${maxQ}_{pred}$ value, lowering the importance of the future reward compared to the immediate reward and θ−are the parameters of the target network (a delayed copy of the prediction network).

The loss function minimized during training is the Mean Squared Error (MSE) between the predicted and target Q-values:

To enhance Q-Value predictions, a Mean Squared Error (MSE) function is employed. MSE quantifies the disparity between predicted Q-values and target Q-values, contributing to the refinement of the learning process.

(2) $$MSE\;Loss=\frac{1}{N}\sum_{i=1}^{N}{\left(Q_{target}-Q_{pred}\right)}^{2}$$

N is the number of samples stored in memory, and the target and predicted value,$Q_{target}$ and $Q_{pred}$, respectively. After each episode of training the target Q-Values for action-state pairs are calculated based on the following equation:

Two neural networks are used, the main Q-network and the target network, with periodic updates from the former to the latter [32,36].

Rewards are based on the time vehicles or pedestrians remain stationary, as can be seen in Equation (1). $wt_{veh,t}/wt_{ped,t}$ is the amount of time in seconds a vehicle or a pedestrian has a speed of less than 0.1 m/s at *t*, since the spawn into the environment. With this metric, the values of *atwt*_t_ does not reset, until the vehicle or pedestrian cross the intersection, *n* represents the total number of vehicles/pedestrians in the environment in *t.* This approach ensures efficient traffic flow and reduced delays.

(3) $$atwt_{veh,t}=\sum_{veh=1}^{n}wt_{\left(veh,t\right)}\;\;atwt_{ped,t}=\sum_{ped=1}^{n}wt_{\left(ped,t\right)}$$

The final reward function, $r_{t},$ is expressed in Equation (2), where *atwt*_t_ and *atwt_t_*_−1_ represent the accumulated total waiting time of all vehicles and pedestrians at the intersection at time steps *t* and *t* − 1, respectively. The parameters *p_veh_* and *p_ped_* denote the assigned weights for vehicles and pedestrians, respectively, and are defined according to the priority levels intended for each agent type during network training. By tuning these weights, the agent can be trained to favor a specific group (e.g., prioritizing vehicular flow over pedestrian crossings), or to maintain a balanced approach when equal importance is desired. Through this reward structure, the agent progressively learns an optimal policy that minimizes waiting times while aligning with the desired traffic management objectives.

(4) $$r_{t}=p_{veh}\left(atwt_{veh,t-1}-atwt_{veh,t}\right)+p_{ped}\left(atwt_{ped,\;t-1}-atwt_{ped,t}\right)$$

The agent’s experiences are stored in a replay memory as tuples ex = (*st*, *at*, *rt*, *st*+1), forming a dataset Dt = (e1, e2, …, et), which is incrementally built as the agent interacts with the environment. During the training process, random mini-batches of experiences are sampled from this buffer to disrupt the temporal correlations between consecutive interactions. This randomization improves learning efficiency and stability by preventing the agent from overfitting to sequential patterns. The replay buffer has a fixed storage capacity, and once this limit is reached, older experiences are systematically replaced by newer ones, ensuring that the dataset remains representative of the agent’s most recent and relevant interactions with the environment.

Different DRL methods suit varying traffic scenarios and are categorized as centralized or decentralized control approaches [37,38,39]. Centralized methods train a global agent to manage the entire network. Individual agents observe intersections and share their experiences to train the global agent, which determines actions for all intersections. Decentralized methods treat multi-intersection control as a multi-agent system. Each agent is trained independently to manage a single intersection, observing and responding only to its local traffic environment

## 4. AI Optimization Model and Rerouting Strategy

The integration of Multi-Agent Reinforcement Learning (MARL) systems into intelligent traffic management enables the coordination of multiple decentralized agents operating in dynamic and partially observable environments. In such systems, each agent, representing, for example, a traffic signal controller, an AGV, or a pedestrian crossing assistant, interacts with its surroundings and learns optimal behaviors through continuous feedback. MARL frameworks are particularly suitable for airport terminal scenarios, where simultaneous decisions must be made at various intersections and pathways, while considering real-time traffic flow, pedestrian density, and route priorities. These agents learn policies not only based on their individual experiences but also by adapting to the behavior of other agents, enabling cooperative or competitive strategies depending on the application. This section outlines the architecture, learning process, and communication mechanisms underpinning MARL deployment within the proposed traffic control model.

### 4.1. Multi-Agent Reinforcement Learning Strategies

#### 4.1.1. Agent Architecture and Interaction Model

In the proposed MARL-based framework, each agent is designed with a perception, decision-making, and communication layer. The perception layer collects environmental observations, such as queue lengths, waiting times, signal phases, and pedestrian density in specific zones. The decision-making layer processes these observations using a policy network, trained via reinforcement learning techniques, to determine the most appropriate action, such as switching traffic signal phases, regulating AGV movements, or granting pedestrian crossing access. Finally, the communication layer facilitates information exchange between agents, enabling coordination and cooperative behavior, particularly at adjacent intersections or in overlapping control zones.

Although each agent operates at a separate intersection, a centralized training approach is adopted. Each agent collects experience tuples (st, at, r_t_, s_t+1_, which are shared and aggregated into a global replay buffer. These experiences are used to train a single shared Q-network. This is possible due to the homogeneity of the intersection configurations, allowing knowledge to be transferred across agents. During inference, each agent uses the shared policy, which has been trained using experiences from all intersections, ensuring coordinated and consistent behavior across the traffic network.

Agents operate asynchronously, reacting to local conditions while considering shared goals such as reducing overall waiting time, minimizing congestion, and maintaining safety. The learning process is inherently decentralized, allowing each agent to independently update its policy based on local observations and reward signals. However, a central coordination mechanism (e.g., a communication manager or centralized critic) may be introduced during training to stabilize learning and align individual policies with system-wide objectives.

To support real-time adaptability, agents employ experience replay buffers, policy updates via deep Q-networks or actor-critic methods, and exploration strategies such as epsilon-greedy or entropy-based exploration. This enables each agent to gradually learn optimal actions under varying traffic patterns and operational conditions.

#### 4.1.2. Practical Agent Interactions in Coordinated Scenarios

To illustrate the potential of the proposed MARL system, consider the following practical interaction scenarios between agents within the airport traffic environment:

- *Coordination Between Adjacent Intersections (I2I)*: Two traffic signal control agents located at adjacent intersections, say T0 and T1, continuously exchange information about queue lengths and approaching traffic flows. For instance, if the agent at T0 detects a long queue forming in the eastbound lane, it can preemptively notify T1, allowing it to adjust its signal timings to avoid downstream congestion. Conversely, T1 can also notify T0 of a sudden influx of vehicles or pedestrians, prompting T0 to delay green phases momentarily to better synchronize flow. Such inter-agent coordination enables smoother traffic progression across multiple intersections, reduces abrupt stops for AGVs, and minimizes the occurrence of blocking situations at crossings.
- *Coordination Between Signal Agents and AGVs (I2D/D2I):* In this scenario, the agent managing a traffic light communicates directly with AGVs approaching a decision point. Suppose an AGV is en route to intersection T2, and the local agent forecasts a high pedestrian density in the area. It can proactively communicate a delay message or suggest an alternative route to the AGV to maintain safety and operational efficiency. Conversely, the AGV, via D2I communication, can request early green phase allocation based on real-time mission urgency or cargo priority, which the traffic signal agent can factor into its decision-making process.
- *Coordination Between Signal Agents and Pedestrians (I2P/P2I)*: Pedestrian agents communicate their intention to cross, including direction and location, via P2I messages. In response, the infrastructure agent provides estimated wait times or the activation schedule for the corresponding crossing phase. For example, if a pedestrian at G3,0 V2,10 signals intent to cross eastbound at TL:14, the agent can evaluate whether to integrate the crossing request into the next cycle based on overall system state. If multiple pedestrian agents request the same crossing, the agent can adjust its policy to minimize cumulative waiting time while balancing AGV flow.
- *Multi-Agent Cooperation in Dynamic Environments:* In complex scenarios—such as during peak passenger arrivals or cargo transfers—agents may dynamically form coalitions to handle localized congestion. For example, agents at T1, T2, and T3 can share aggregated states and collaboratively adjust their policies, prioritizing evacuation routes or accelerating AGV delivery pathways. This emergent cooperative behavior, fostered by MARL, enhances scalability and resilience, particularly in unpredictable environments.

#### 4.1.3. Centralized Traffic Management with Multi-Agent Reinforcement Learning: Routing Strategies and Scenario Analysis

Building upon the architecture described, this part focuses on the centralized coordination strategy adopted for traffic control. To enhance overall efficiency and adaptability, a global learning agent is introduced, capable of handling diverse traffic patterns by integrating experience from multiple local agents. The following details the methodology, routing strategies, and the various traffic scenarios analyzed during system evaluation.

**Figure 8:**
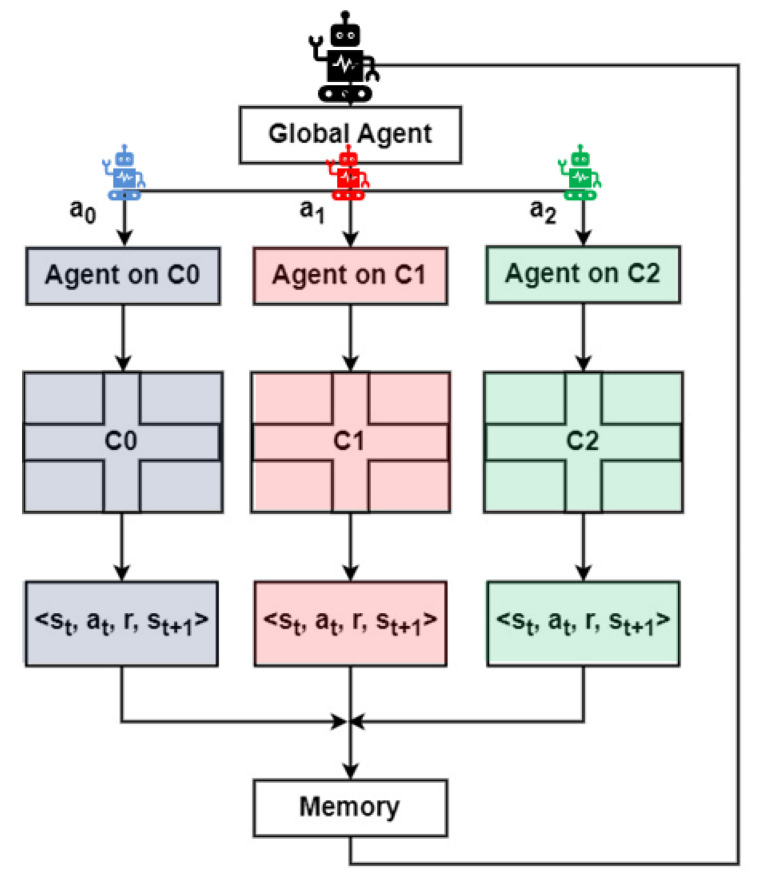
A schematic of the algorithm using centralized MARL.

The adopted method follows a centralized approach, as illustrated in Figure 8, where individual agents share their experiences to train a global agent that controls all intersections.

Traffic dynamics were evaluated across single-, two-, and three-junction configurations. Given the homogeneity of the intersections, the observations collected by each local agent are used to train a single neural network, which functions as the global agent. This approach has proven effective in managing both pedestrian and vehicular flows. However, occasional queue peaks may still occur due to the global agent’s limited awareness of neighboring queue conditions.

To address this limitation, queue length threshold values were integrated, allowing for the regulation of critical sections based on intersection capacity [28,40]. Three distinct traffic scenarios were considered to reflect varying conditions and routing strategies:

1. *Standard Scenario:* 75% of vehicles travel straight, while 25% make turns.
2. Symmetric Rerouting Scenario: Triggered under high traffic demand or incidents, 75% of vehicles are redirected through turning maneuvers to balance flow across all directions.
3. *Asymmetric Rerouting Scenario*: Applied in situations where traffic flow at one intersection significantly affects neighboring junctions. In this case, the reward structure is adapted to prioritize global traffic efficiency by favoring specific directions.

The system begins with the standard scenario and dynamically switches to symmetric rerouting when high congestion levels are detected, ensuring a more balanced distribution of traffic. In more critical situations, asymmetric rerouting is activated to prioritize key routes and alleviate network-wide congestion.

Rerouting and anti-bottlenecking techniques dynamically adjust control strategies based on congestion thresholds. While the neural network is centrally trained, agents locally implement rerouting strategies, adapting to optimize traffic flow even under heavy demand, ensuring efficient and balanced operations.

Based on the study conducted across various scenarios, we gained insights into traffic queue behavior and identified the capacity limits for each lane. To mitigate the absence of direct information exchange between agents, we integrated this prior knowledge into the network by establishing threshold values for the queues. This allows the global system to regulate traffic flow in critical sections of an artery, evaluating the volume of traffic that can be accommodated in each direction.

#### 4.1.4. Cooperative Reward Mechanisms

In MARL environments, the design of the reward function and the adopted learning strategy play a crucial role in ensuring effective coordination and convergence towards optimal global behavior. By promoting collaboration among agents, a global or shared reward structure can be implemented, where each agent’s individual reward incorporates not only local performance metrics but also system-level objectives. This helps mitigate selfish behaviors that could otherwise result in suboptimal outcomes for the overall traffic system.

For instance, the reward function $r_{t}$ can be composed of a weighted sum of:

- Local waiting time reduction at a given intersection;
- Queue balancing across neighboring intersections;
- Pedestrian comfort metrics, such as average waiting time or number of successful crossings per cycle.

Such a reward structure encourages agents to consider both local optimization and network-wide impact, fostering global convergence.

To further optimize traffic in rerouting scenarios, upstream anti-bottlenecking and smart rerouting techniques are employed, adjusting intersection control in real-time based on congestion levels and dynamically assigning priority to alternative routes. To integrate the three traffic scenarios (“standard”, “symmetrical rerouting”, and “asymmetrical rerouting”) into the reward equation (Equation (4)), the reward was adjusted dynamically to account for the specific goals and constraints of each scenario. This was achieved by introducing scenario-specific weights, ω, that adjusts the priority of the reward based on the current traffic scenario or parameters into the reward function, ensuring that the policy aligns with the objectives of each traffic condition. Below is a modified version of the reward equation:

(5) $$r_{t}=\omega (p_{veh}\left(atwt_{veh,t-1}-atwt_{veh,t}\right)+p_{ped}\left(atwt_{ped,t-1}-atwt_{ped,t}\right))$$

In the standard scenario ω = 1 (baseline weight for normal traffic flow), $p_{veh}$ and $p_{ped}$ are set to reflect a balance between vehicles and pedestrians. In the symmetrical scenario ω > 1 to increase the system’s responsiveness to congestion. $p_{veh}$ is adjusted to prioritize turning movements to redistribute traffic effectively, potentially reducing delays caused by oversaturated straight-through lanes. In the asymmetrical rerouting scenario, ω is dynamically set based on both directions’ priorities (for instance, ω = 1.5 on the prioritized direction and ω = 0.8 for the less prioritized one and $p_{veh}$ is weighted heavily in the prioritized direction to reflect its importance.

### 4.2. Simulation Environment and Validation

**Figure 9:**
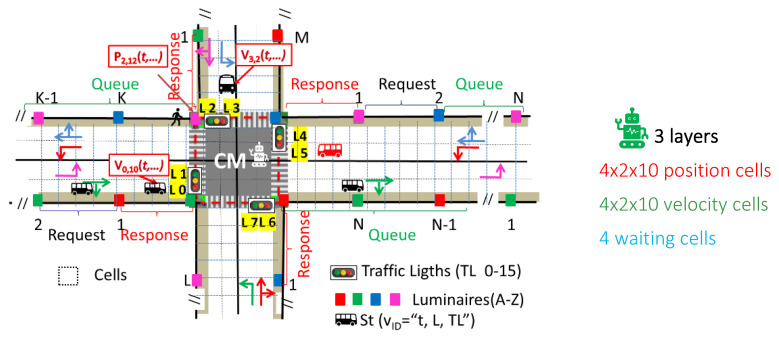
A schematic of the state representation for each junction. The arrows are assigned to the traffic directions.

The simulation environment was designed to reflect realistic traffic conditions, using a scenario with three connected four-arm intersections, each with two lanes in both directions (Figure 3). Each intersection is controlled by an individual agent, which maps the surrounding environment into a grid-based representation to collect information on both vehicular and pedestrian traffic (Figure 9). This enables the agents to perceive traffic dynamics in their vicinity and support intelligent decision-making.

Each intersection is structured into three observation layers, comprising a total of 164 cells:

- *Layer 1* consists of 80 binary cells (10 per lane), indicating vehicle presence. A value of ‘1’ represents an occupied cell, while ‘0’ indicates an empty cell.
- *Layer 2* mirrors the structure of the first layer and encodes the normalized speed of vehicles in each corresponding cell, if present.
- *Layer 3* includes 4 cells representing pedestrian waiting zones, capturing the number of pedestrians waiting for their crossing phase to become active.

This layered state representation allows each agent to develop a detailed spatial and dynamic understanding of its intersection. Moreover, it closely aligns with the state information perceived via Visible Light Communication (VLC), as illustrated in Figure 9. Vehicles are identified over time by the lane they occupy and the associated traffic light signals, while pedestrians are recognized based on the waiting zone and their interaction with the corresponding traffic light.

To assess the effectiveness of the proposed V-VLC system in multi-terminal airport environments, simulations were conducted using the Simulation of Urban Mobility (SUMO), an open-source microscopic traffic simulation platform [41,42,43,44]. SUMO enables the modeling of vehicles, public transportation, and pedestrian movements, with customizable parameters such as traffic density, vehicle types, and network layouts. This allows for thorough testing and validation of traffic control algorithms aimed at optimizing flow, managing intersections, and improving pedestrian crossings.

The SUMO API provides interfaces to external programs, facilitating real-time extraction of traffic flow statistics and enabling interaction with the agent-based control logic. State and phase diagrams further support the evaluation of traffic signal dynamics. Within this simulation framework, agents iteratively learn and refine traffic light control policies to enhance overall efficiency across intersections, sidewalks, and lanes.

Pedestrians are introduced into the simulation environment with a walking speed of approximately 1 m/s (3.6 km/h), consistent with average real-world pedestrian velocities. This information is incorporated into the SUMO incentive mechanism, guiding the learning process of the agents to optimize traffic distribution, reduce vehicle and pedestrian waiting times, and improve green light utilization, ultimately enhancing system-wide performance.

These simulation settings provide a realistic and controlled environment to evaluate the performance of the proposed traffic management approach. By combining detailed state representations, real-world pedestrian behavior, and the flexibility of SUMO, the system enables robust validation of multi-agent control strategies under diverse traffic conditions. This ensures that the trained agents can effectively respond to dynamic scenarios and contribute to the seamless coordination of vehicle and pedestrian flows in complex environments such as multi-terminal airports.

### 4.3. Rerouting Techniques: Network Training and Testing

To compare the effectiveness of different rerouting strategies, three neural networks were independently trained using a reward function with equal weighting, 50% for autonomous vehicles and 50% for pedestrians. This balanced approach ensures that both mobility aspects are considered during the learning process.

**Figure 10:**
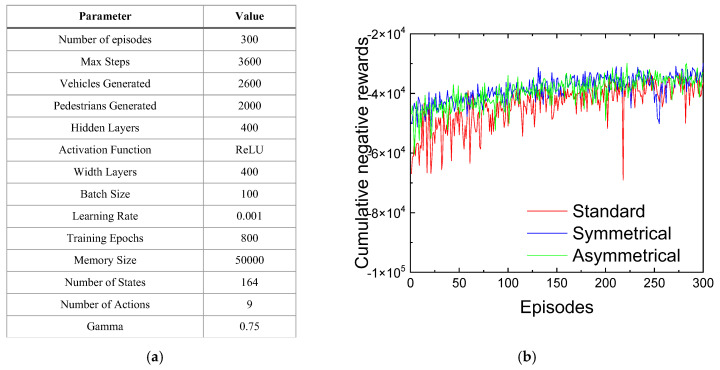
(**a**) Network training parameters. (**b**) Cumulative negative reward curves over training episodes, comparing the performance of the three rerouting strategies.

The simulation environment comprised 2600 vehicles and 2000 pedestrians, distributed across 300 episodes, each lasting 3600 s. The key training parameters used during the experiments are summarized in Figure 10a.

Figure 10b illustrates the cumulative negative rewards obtained throughout the training process, enabling the comparison of learning efficiency across the three neural networks. The reward curves show a generally positive slope over the episodes, indicating a steady improvement in agent performance. Although some fluctuations are observed, mainly due to variations in traffic scenarios during training, the system successfully adapts to these dynamics, resulting in effective and robust network learning.

In the standard scenario, the higher bidirectional traffic flow leads to lower rewards due to increased congestion, extended waiting times, and larger queues at junctions. Conversely, both rerouting scenarios enhance rewards by dynamically suggesting optimal routes, effectively reducing queues and alleviating congestion at critical intersections. Prioritizing turning options or optimizing east-to-west traffic flow in rerouting scenarios further decreases intersection pressure, contributing to higher rewards. Overall, the symmetric and asymmetric rerouting approaches demonstrated faster convergence, greater stability, and more consistent reward patterns compared to the standard arterial scenario.

The traffic environment consists of three terminals, as previously noted, which are unevenly spaced. The distance between T0 and T1 is 400 m, while the distance between T1 and T2 is 200 m. Positioned between two terminals, T1 is regarded as the most critical of the three since it must handle traffic flows from both T0 and T2. This places additional demands on the network to prioritize traffic management at this intersection. Inefficient management at T1 could disrupt airport operations, resulting in longer waiting times and increased queues for both pedestrians and vehicles.

**Figure 11:**
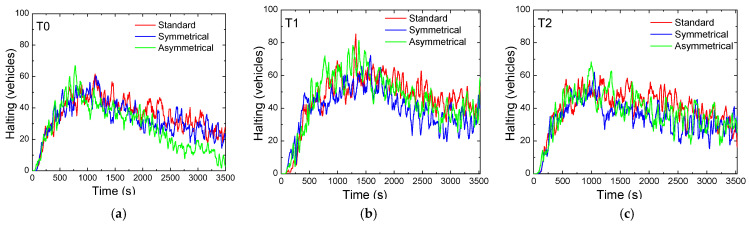
Comparison of vehicle halting trends over time at intersections under standard, symmetric rerouting, and asymmetric rerouting scenarios: (**a**) Terminal T0, (**b**) Terminal T1, and (**c**) Terminal T2.

Figure 11a–c illustrate the number of stopped vehicles (halting) at each intersection across the three different scenarios. For all three terminals, the standard scenario consistently exhibits the highest number of stopped cars compared to the other scenarios.

The rerouting symmetric and asymmetric scenarios utilize advanced control techniques and occupancy detection mechanisms to redirect vehicles, thereby preventing terminals from becoming overwhelmed

Traffic is guided toward less congested routes, alleviating pressure on critical areas. On the stretch between T0 and T1, the maximum allowable number of stopped cars is approximately 25, while between T1 and T2, this limit is around 10 vehicles. When these thresholds are reached, micro-control strategies are implemented at the terminals to restrict additional vehicles from accessing these routes. These strategies include activating traffic signal phases that block entry to these areas or initiating pedestrian phases to manage congestion. Among the three terminals, T1 consistently registers the highest number of stopped vehicles. This observation aligns with previous analyses, confirming T1’s role as the critical terminal responsible for managing traffic from both neighboring intersections.

**Figure 12:**
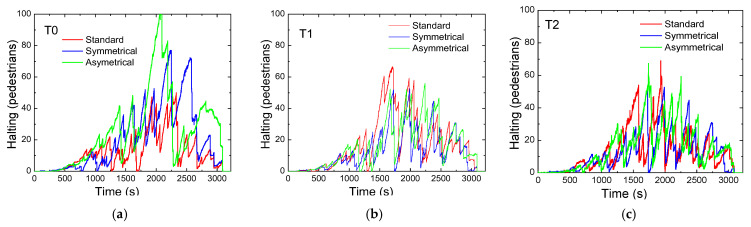
Comparison of pedestrian halting trends over time at intersections under standard, symmetric rerouting, and asymmetric rerouting scenarios: (**a**) Terminal T0, (**b**) Terminal T1, and (**c**) Terminal T2.

Analyzing the case of pedestrians shown in Figure 12a–c, the graphs illustrate pedestrians waiting in designated areas for different scenarios at each terminal. It is evident that, across the three terminals and various scenarios, there are distinct peaks in the number of stationary pedestrians, followed by abrupt drops. This pattern suggests the activation of a pedestrian phase. These peaks could be attributed to a higher flow of pedestrians at specific intersections or, alternatively, to a significant volume of vehicular traffic, prompting the network to prioritize vehicles temporarily, thereby delaying the activation of the pedestrian phase and causing pedestrians to remain in waiting zones for longer periods. Pedestrian traffic management must be carefully executed to ensure the smooth flow of vehicles while safeguarding pedestrian safety. During training, the networks learn when to activate a pedestrian phase by assessing, through the reward obtained, whether the activation would excessively disrupt vehicular traffic. Overall, the rerouting scenarios maintain pedestrian flow and safety throughout the testing period, demonstrating its ability to balance vehicle and pedestrian needs.

### 4.4. Global Agent Decisions

The spacing between terminals plays a significant role in traffic management within this environment. The 400 m stretch between T0 and T1 provides a larger vehicle storage capacity, effectively acting as a buffer. Careful management of green light phases and their durations is essential to prevent vehicles waiting in this 400 m zone from completely filling the shorter 200 m stretch between T1 and T2, which would impose excessive strain on terminal T2. This highlights the critical importance of efficient traffic control at terminal T1.

**Figure 13:**
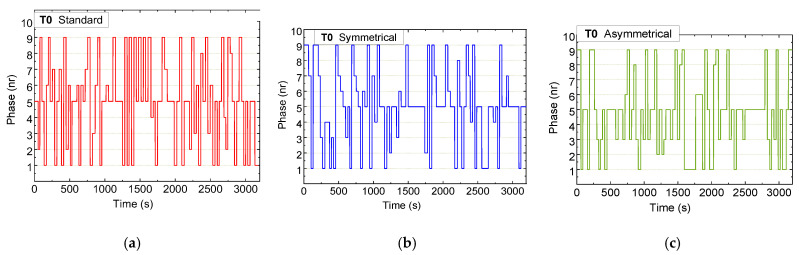
Temporal comparison of active phases (agent actions) at Terminal T0 across three scenarios: (**a**) Standard scenario. (**b**) Symmetric rerouting scenario. (**c**) Asymmetric rerouting scenario.

**Figure 14:**
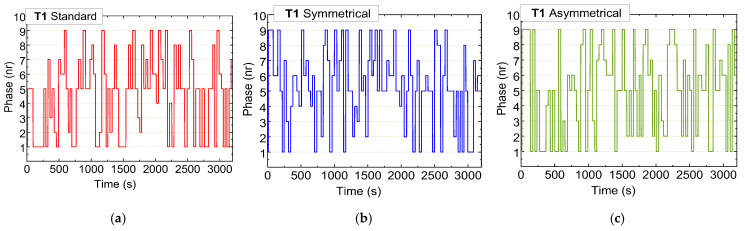
Temporal comparison of active phases (agent actions) at Terminal T1 across three scenarios: (**a**) Standard scenario. (**b**) Symmetric rerouting scenario. (**c**) Asymmetric rerouting scenario.

**Figure 15:**
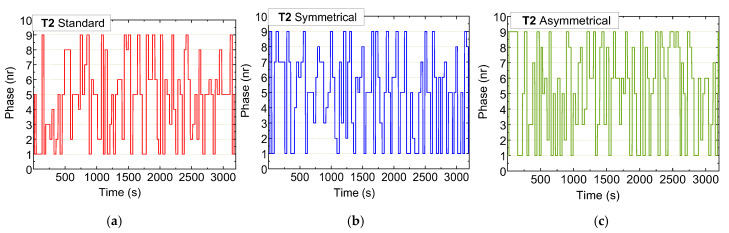
Temporal comparison of active phases (agent actions) at Terminal T2 across three scenarios: (**a**) Standard scenario. (**b**) Symmetric rerouting scenario. (**c**) Asymmetric rerouting scenario.

Figure 13, Figure 14 and Figure 15 illustrate the sequential trends of active phases at intersections T0, T1, and T2, respectively, over a one-hour testing period across three scenarios: standard (a), symmetrical rerouting (b), and asymmetrical rerouting (c).

Unlike fixed-phase systems, the adaptive system dynamically responds to real-time conditions, activating pedestrian and AGV phases only when required, thus optimizing phase utilization. In the standard scenario, traffic management relies heavily on traditional phase activation, prioritizing vehicle movement. This results in less-flexible congestion management, especially at critical intersections such as T1 and T2.

In contrast, the rerouting scenarios employ micro-control strategies that dynamically adjust phase activations based on traffic demands. These strategies place a particular emphasis on reducing congestion at T1 and T2 by redistributing traffic and preventing terminals from becoming overloaded. The rerouting scenarios also demonstrate greater efficiency in managing pedestrian phases by prioritizing vehicle traffic when necessary, leading to smoother traffic flow.

Overall, the rerouting scenarios exhibit more dynamic and balanced phase utilization. By redistributing traffic loads and optimizing available capacity, particularly at T1 and T2, they achieve better congestion management compared to the standard scenario.

### 4.5. Comparative Analysis of Standard and Rerouting Scenarios: Activations and Traffic Management Strategies

This section evaluates the differences between the *standard* and *symmetric rerouting* scenarios in terms of phase activations and traffic management strategies.

**Figure 16:**
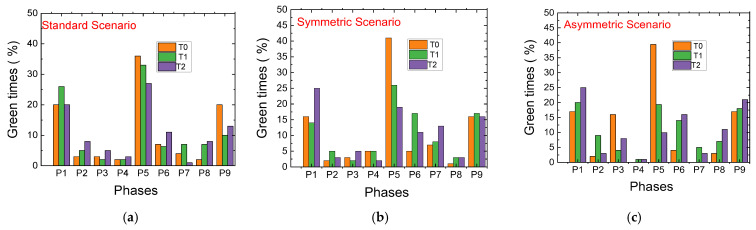
Temporal comparison of green time trends across all active phases at intersections T0, T1, and T2. Active phases are shown at the top for clarity: (**a**) Standard scenario. (**b**) Symmetric rerouting scenario. (**c**) Asymmetric rerouting scenario.

Table 2 presents the overall percentages of green times for intersections T0, T1, and T2 over a training segment for the standard and regrouting’s scenarios. These values are also visualized in Figure 16 for comparison.

*Phase 1 (N-S): Traffic Flow Control*
  - Standard Scenario: Activated approximately 20% of the time, particularly increasing at Terminal T1 to prevent overload at Terminal T2.
  - Rerouting Scenario: Displays a 5% reduction in activation at the first two terminals, while increasing activation at T2. This prioritization helps manage traffic flow along the critical 200 m stretch between T1 and T2, dynamically balancing traffic demands.
*Phase 5 (W-E): Vehicle Storage and Congestion Management*
  - Standard Scenario: Activated around 30% of the time, with T0 seeing the highest activation due to the larger 400 m stretch suitable for vehicle storage. Activation drops significantly at T1 and T2, where the shorter 200 m distance requires stricter congestion control.
  - Symmetric Rerouting Scenario: Shows a 20% reduction in activation at T1 and T2, reflecting measures to limit vehicle flow to 15 vehicles on the shorter stretch. This highlights rerouting strategies that emphasize congestion management.
  - Asymmetric Rerouting Scenario: Prioritizes E > W traffic. At T0, activation increases to 40%, efficiently clearing vehicles from the 400 m road. At T1 and T2, activation drops to 20% and 10%, reflecting greater caution to maintain the 15-vehicle limit on shorter roads.
*Phase P9: Pedestrian Flow Optimization*
  - Standard Scenario: Pedestrian phases are activated 10–20% of the time, with T0 having the highest rate at 20%, leading to higher pedestrian accumulation.
  - Rerouting Scenarios: Pedestrian phase activation decreases at T0 and increases at other terminals, prioritizing vehicle flow while optimizing pedestrian navigation.
*Phase 6: Clearing Vehicles on 200 m Stretches*
  - Standard Scenario: Activated 13% of the time at T2 to maintain smooth traffic flow from the 400 m stretch.
  - Rerouting Scenarios: Activation slightly increases at T1 and T2 through micro-control measures, reducing vehicle buildup and improving flow with rerouting strategies.
*Phase 7: Opposite Direction Traffic Management*
  - Symmetric Rerouting Scenario: Activation increases to complement rerouting strategies, ensuring balanced traffic flow across intersections and enhancing network-wide performance.

The rerouting scenarios introduce *dynamic traffic control* strategies that adjust to changing traffic conditions, enabling the following:

- Reduced congestion through micro-control and rerouting strategies.
- Balanced flow across intersections by prioritizing directions or redistributing traffic.
- Improved pedestrian flow optimization without sacrificing vehicle throughput.
- Enhanced management of shorter roads using vehicle limits and activation thresholds.

These results highlight the effectiveness of rerouting and micro-control approaches in managing complex traffic dynamics within multi-terminal airport environments.

## 5. Adaptive Reward Mechanism with Horizontal and Vertical Shared Arteries and Pedestrian Integration

### 5.1. Environment Mapping

**Figure 17:**
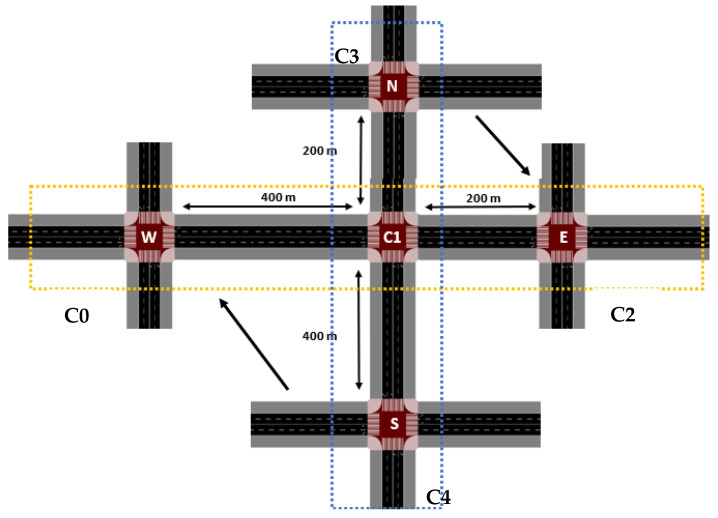
New traffic environment consisting of five intersections (C0-4) and two different arteries (W-E and N-S).

In this section, the environment has been expanded to include five intersections, introducing a new level of complexity and realism. As illustrated in Figure 17, the environment now consists of two main arteries—one horizontal and one vertical—connected by shared intersections. The horizontal artery is formed by intersections W–C1–E, and the vertical artery by N–C1–S. Importantly, intersection C1 plays a central role in this new setup, as it connects the two arteries and serves as a critical point for traffic flow management.

The new environment design builds upon the original setup (Figure 3a), which was limited to just three horizontal terminals: W, C1, and E. In that environment, a high-traffic scenario and three control strategies (standard, rerouting, and asymmetric rerouting) were studied, allowing for a deep understanding of how these intersections operated when they formed a single artery. In this section, the focus shifts to the more complex system of two arteries, with C1 at the intersection of both. As a result, this central terminal (C1) will be analyzed in more detail to assess how it affects the overall traffic dynamics.

In this updated environment, intersection C1 no longer has independent entries; instead, its traffic is entirely influenced by the vehicles and pedestrians coming from the four neighboring terminals. These vehicles flow into C1’s lanes based on the phase activation decisions made by other C0, C2, C3, and C4 agents in the system. Understanding the impact of C1 and its role in the traffic flow will be a key objective, as it is expected to play a significant role in ensuring smooth traffic management.

In terms of traffic management, the previously implemented strategies, such as rerouting and asymmetric rerouting, will still be applicable. Moreover, the influence of these strategies on the final reward will also be assessed, as the system continues to optimize traffic flow through the five intersections.

As a result, a key question arises: Should intersection C1 be considered *a global agent*, an agent responsible for managing the flow of traffic from neighboring intersections and ensuring overall traffic smoothness as in Figure 8? This question will be examined in the study to determine whether C1 can be treated as the central control point for the system or if the system should adopt a more distributed approach to traffic management.

### 5.2. Environment Mapping Strategy

For this study, the environment has been carefully modified, but the core principles remain the same. Each of the five intersections in the environment is designed to be homogeneous, ensuring that agents have equal training experiences despite the expanded complexity. This is crucial for maintaining a consistent learning process where agents can share knowledge through a collective memory (Figure 8), rather than requiring each agent to learn independently at each intersection.

During the neural network training process, each agent’s experience, denoted as *e_x_ = (s_t_*, *a_t_*, *r_t_*, *s_t_*_+1_*),* is stored in a shared memory. Here, *st* represents the state, at the action, *r_t_* the reward, and *s_t_*_+1_ the subsequent state. By storing these experiences, agents can later sample from the shared memory to train the neural network. This approach allows the system to take advantage of the collective learning experiences of all agents, improving the overall performance.

To facilitate this shared experience across different intersections, the system treats each intersection as equivalent, with adjustments made to account for their differing orientations in the environment. This is achieved by rotating intersections, ensuring that all agents perceive them as having the same structure and traffic flow dynamics. This mapping strategy ensures that the environment is treated as a cohesive system, where agents can seamlessly learn from one intersection to another without requiring unique training for each individual location.

### 5.3. Adaptive Reward Mechanism

Given the complexity of airport environments, where vehicular traffic (e.g., shuttle buses, service vehicles) and pedestrian movement (e.g., passengers, airport personnel) coexist, the reward structure within RL (Equation (3)) must be carefully designed to balance flow efficiency with safety and responsiveness.

Previously, we considered a reward where the weights of both vehicles and pedestrians were 50%/50%, respectively, thus keeping the system balanced. Now the intention is to study a scenario in which vehicles need to be prioritized, so the weights in the reward have been altered to reflect this desired priority. Considering this, a weight distribution of 75% for vehicles and 25% for pedestrians was applied, reflecting the operational need to prioritize vehicular movement in airport logistics. However, this can lead to suboptimal pedestrian treatment if not properly regulated.

A common issue observed during training was the tendency of agents to continuously select strong vehicular phases (e.g., north–south (P1) or west–east (P5) flows) while neglecting pedestrian phases (P9) in the standard scenario (Figure 16a). This behavior, if left unchecked, results in an accumulation of waiting pedestrians, increasing the risk of non-compliance with traffic signals (e.g., crossing during red phases), ultimately jeopardizing overall safety (Figure 12).

To promote a more balanced phase activation strategy, a penalty-based reward adjustment mechanism was introduced. This mechanism penalizes the learning agents when pedestrian phases are not activated within a predefined temporal window, specifically after four consecutive vehicular phase activations. If no pedestrian phase is triggered within this interval, a penalty term (pen = 0.5) is incorporated into the reward function. Conversely, if pedestrian phases are activated with adequate regularity, the penalty is neutralized (pen = 0).

This adaptive strategy enables dynamic reweighting of the reward structure, temporarily shifting the relative priority from the default 75% vehicular, 25% pedestrian weighting to 25% vehicular, 75% pedestrian, thus encouraging the system to respond to pedestrian demands without compromising overall traffic efficiency. The penalty-driven adjustment guides the network toward a more inclusive phase selection behavior, ensuring that pedestrian needs are adequately addressed while preserving effective vehicular flow management.

The adapted reward function is defined as follows:

(6) $$r_{t}=\left({awt}_{t-1}-{awt}_{t}\right){.w.(p}_{veh}-pen)+\left({awt}_{t-1}-{awt}_{t}\right).w.{(p}_{ped}+pen)$$

where *awtₜ* represents the average waiting time at time *t*, and *w* denotes a scaling factor. This approach encourages the agent to avoid prolonged neglect of pedestrian phases, promoting a fairer distribution of green phases across user types.

### 5.4. Performance Evaluation

Two neural networks were trained under identical simulation conditions—a low-traffic scenario comprising 1800 vehicles and 2000 pedestrians—differing only in the inclusion or exclusion of a penalty term within the reward structure. The training results showed convergence in both networks, with progressively less negative cumulative reward curves, indicating consistent learning progression across episodes.

**Figure 18:**
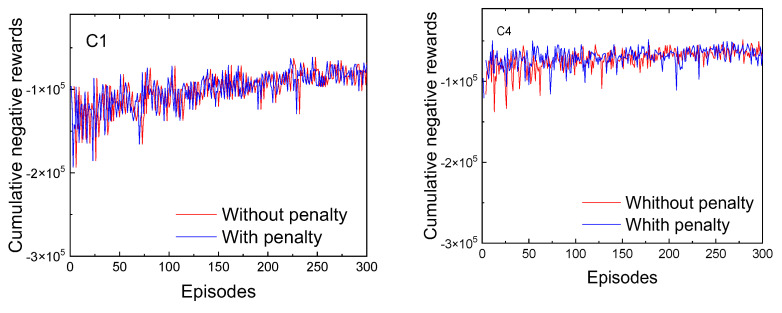
Cumulative negative rewards with and without penalty at intersections C1 and C4.

Figure 18 illustrates the cumulative negative rewards obtained during training at two of the five intersections (C1 and C4). It can be observed that both networks exhibit balanced behavior; however, the reward curve becomes less negative more rapidly at intersection C4 than at C1, indicating a faster learning rate in that scenario, as expected.

During the testing phase, each network was evaluated across multiple simulation episodes, with performance metrics such as pedestrian halting time, vehicle halting time, and average traffic speed being recorded. The testing consisted of three simulation episodes, from which the relevant environmental outputs were collected and used to compute average values per intersection.

**Figure 19:**
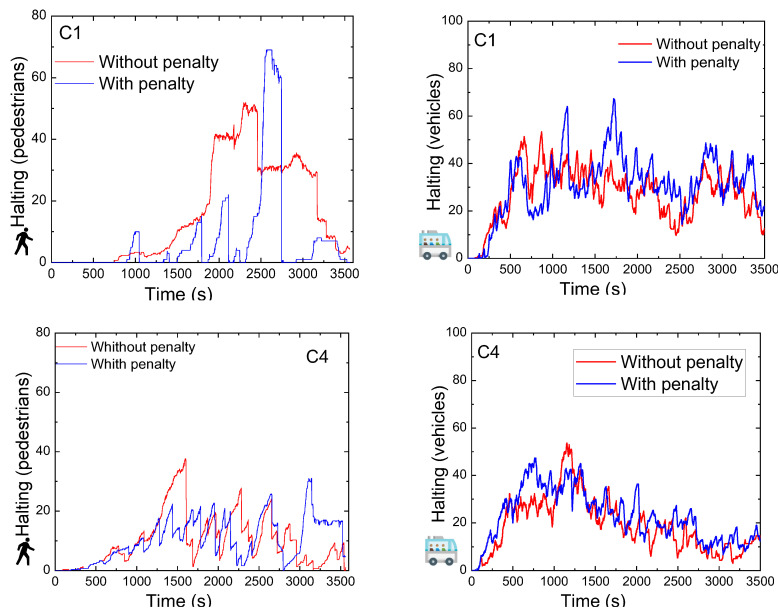
Comparison of trends over time for pedestrian and vehicle halting sessions at intersections C1 and C4 with and without penalties.

Figure 19 presents a time-based comparison of pedestrian and vehicle halting events at intersection C1 and C4, with and without the application of the penalty term. The principal objective of this evaluation is to determine whether incorporating a penalty in the reward structure results in more frequent activation of pedestrian crossing phases, thereby reducing pedestrian waiting times. The results clearly show that the network trained with reward penalization achieved much lower pedestrian halting times compared to the network without penalization, especially at C1 where without penalty they remain blocked. This indicates improved responsiveness to pedestrian needs.

Notably, vehicle-related metrics remained consistent across both networks, confirming that the integration of the penalty term did not compromise vehicular flow efficiency. These results demonstrate that the network can effectively manage both pedestrian and vehicle traffic. The reward penalty mechanism successfully guides the network to activate pedestrian phases more regularly while maintaining appropriate prioritization for vehicles.

**Figure 20:**
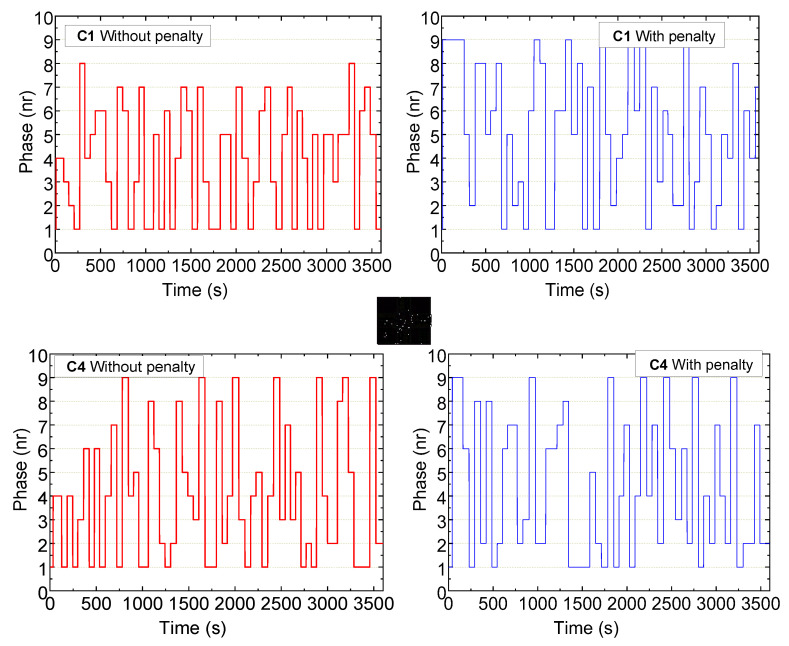
Comparative analysis of active phase trends (agent actions) at intersections C1 and C4 over time, with and without reward penalization.

Figure 20 presents the temporal evolution of active traffic signal phases at intersections C1 and C4 over a one-hour testing period, comparing the system’s behavior under two configurations: without reward penalization (vehicle-prioritized, 75–25%) and with reward penalization (pedestrian-prioritized, 25–75%).

The adaptive traffic management system exhibits real-time responsiveness by selectively activating vehicle and pedestrian phases based on traffic conditions, thereby improving overall phase efficiency. In the non-penalized configuration, the system adopts a conventional control strategy, heavily prioritizing vehicular flow. This is reflected in reduced flexibility in phase scheduling, most notably at C1, resulting in underutilization of available green time and increased pedestrian waiting time.

In contrast, the penalized reward scenario introduces a more balanced and inclusive phase distribution. The system proactively adapts to pedestrian presence and flow intensity, dynamically activating pedestrian phases when needed. This leads to an increase in phase diversity at C1 and an improvement in average congestion clearance times at both intersections. Additionally, pedestrian crossing delays are reduced, ensuring a safer and more human-centric flow management strategy.

Overall, the introduction of reward penalization results in more efficient use of traffic phases, enhanced congestion management capabilities, and improved responsiveness to mixed traffic scenarios—highlighting the benefit of integrating adaptive reward mechanisms in AI-driven airport traffic control systems.

**Figure 21:**
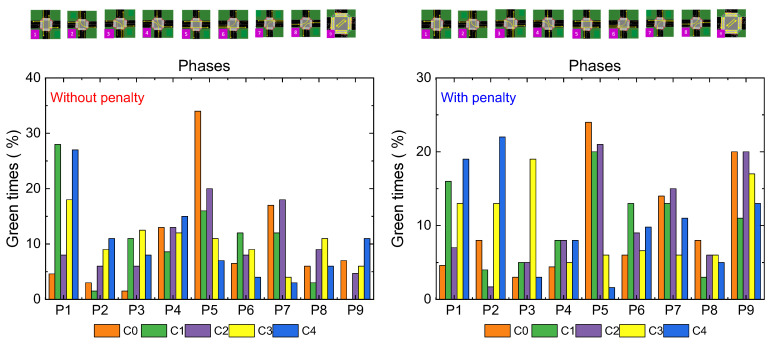
Temporal comparison of green time trends across all active phases at intersections C1 and C4. Active phases are indicated at the top for clarity.

Figure 21 illustrates the sequential activation patterns of traffic signal phases at intersections C1 and C4 over a one-hour testing period, comparing scenarios with and without the application of reward penalization.

- Phase 1 (N-S): Traffic Flow Control

This phase is crucial for maintaining longitudinal flow along the primary axes of the network.

*Without penalization*, Phase 1 shows high green time allocation, particularly at C1 (28%) and C4 (27%), indicating a strong emphasis on maintaining uninterrupted north–south vehicle flow.
*With penalization*, there is a significant reduction in green time at C1 (from 28% to 16%) and at C4 (from 27% to 19%), which allowed for more balanced signal timing and increased allocation to pedestrian phases.
At C0 and C3 (located 400 m from C1), green time for Phase 1 remains stable (~4.6%) in both scenarios, which is expected as these intersections primarily react to local traffic demand rather than being central nodes in the control logic.

- Phase 5 (W-E): Vehicle Storage and Congestion Management

This phase helps clear congestion on transversal lanes and acts as a pressure-relief mechanism.

*Without penalization*, Phase 5 dominates green time at C0 (34%) and is substantial at C2 (20%), reflecting the need to handle side traffic and vehicle accumulation.
*With penalization*, a marked decrease is observed in C0 (from 34% to 24%) and especially in C4 (from 7% to 1.6%), suggesting that this time was reallocated to address pedestrian traffic needs.
Interestingly, at C1, Phase 5 increases from 16% to 20%, likely compensating for the increased activation of pedestrian phases by providing additional clearance time for transversal vehicle flow. This adjustment reflects the system’s ability to rebalance traffic dynamics under new constraints.

- Phase 6: Clearing Vehicles on 200-Meter Segments

This phase is essential for clearing short road segments between intersections, particularly between C1–C2 and C1–C4, which are only 200 m apart.

*Without penalization*, green time allocation ranges from 8% to 12%, with C1 at 12%, highlighting the need to maintain smooth transitions across short links.
*With penalization*, green time in C1 slightly increases to 13% and remains stable in C2 (from 8% to 9%), confirming that even with a shift toward pedestrian prioritization, the system still ensures effective clearing of short-distance vehicle flows.
This result reinforces that network responsiveness to short-segment congestion is preserved under the penalized reward scheme, avoiding traffic buildup in high-density zones.

- Phase 7: Opposite Direction Traffic Management

This phase is vital for balancing traffic in the opposite direction, especially at intersections with turning flows or bidirectional congestion, such **as** C1–C2 segments.

*Without penalization*, Phase 7 maintains moderate but consistent green time allocation, particularly at C0 (17%), C1 (12%), and C2 (18%).
*With penalization*, green time in C1 increases slightly to 13%, and remains solid in C2 (15%), reflecting the system’s capacity to adaptively maintain balanced flow in opposite directions.

A slight reduction at C0 (from 17% to 14%) likely reflects dynamic redistribution in favor of critical pedestrian phases without compromising overall bidirectional flow integrity.

The comparison reveals that reward penalization allows for a more balanced and intelligent redistribution of green time across phases, particularly enhancing pedestrian accessibility while preserving or slightly adjusting key vehicular control phases.

- Phase 1 and Phase 5 experience notable reductions, enabling more responsive pedestrian management.
- Phase 6 and Phase 7 are maintained or slightly enhanced, ensuring no adverse impact on short-distance flow or bidirectional traffic control, particularly critical given the proximity of intersections (200–400 m).

This reflects the adaptive intelligence of the system, which optimizes phase utilization while accommodating dynamic traffic needs, demonstrating the value of integrating a penalization mechanism in the reward structure.

Table 3 presents the percentage of green light allocation for selected traffic phases (1, 5, 6, and 7) at each intersection (C0 to C4), comparing scenarios with and without pedestrian penalization in the reward function. The results highlight the system’s adaptive behavior in reallocating time to balance vehicle flow and pedestrian needs

### 5.5. Implications for Airport Environments

High-density environments such as airports demand a seamless integration of vehicular logistics and pedestrian safety. The proposed adaptive reward model, in conjunction with the VLC-based intelligent traffic control infrastructure, offers a robust solution for managing complex multimodal flows. By enabling dynamic prioritization of pedestrian phases based on real-time learning and contextual awareness, the system promotes a more balanced, responsive, and human-centric traffic management approach.

This adaptability is particularly valuable in airport ecosystems, where pedestrian movement often fluctuates with flight schedules and terminal operations. The system’s capacity to intelligently adjust signal phases ensures enhanced safety for pedestrians without compromising vehicular efficiency. Ultimately, this integration supports a more fluid and efficient coexistence between transportation modalities, contributing to improved operational flow and user experience in airport environments.

### 5.6. Main Challenges Expected During Real-World Deployment

Although the current experimental validation is exclusively based on simulations performed with the SUMO traffic simulator, the system is designed with real-world applicability in mind. This section outlines the main challenges anticipated during real-world deployment and discusses potential mitigation strategies.

- VLC Interference in High-Luminance Environments

One of the primary challenges for VLC-based communication in real-world scenarios is signal degradation due to high levels of ambient light, particularly from natural sunlight in open or glass-rich terminal environments. To address this, the proposed system employs the periodic transmission of signal calibration curves, which allow for real-time adjustment of both transmission and reception parameters in response to changing light conditions. Additionally, error detection and correction mechanisms are integrated into the communication protocol to ensure reliable decoding, even under suboptimal luminance conditions, thereby maintaining the integrity and robustness of the VLC signal.

- Installation Constraints in Large Terminal Spaces

Large indoor environments such as airports present logistical challenges for the deployment of additional communication infrastructure. However, the proposed system leverages existing LED lighting infrastructure, requiring only minimal retrofitting. Specifically, VLC-enabled luminaires and photodetectors mounted on AGVs are sufficient to implement the communication framework, enabling multiple interaction modes (Vehicle-to-Infrastructure, Infrastructure-to-Vehicle, Vehicle-to-Vehicle, and Light-to-Vehicle). This reduces deployment costs and complexity, while ensuring system scalability across wide indoor areas with minimal disruption to existing operations.

- Real-Time Response Delays in Pedestrian–Vehicle Interactions

In real-world environments, timely coordination between AGVs and pedestrians is essential to ensure safety and efficiency. The system addresses this challenge by implementing exclusive traffic phases for AGVs and pedestrians, thereby eliminating conflicts at shared crossings. During an AGV-only phase, pedestrian movement is temporarily restricted to designated waiting areas, and vice versa. This time-separated coordination strategy avoids the need for ultra-low-latency real-time collision avoidance systems and ensures predictable, safe interactions. Consequently, minor response delays do not significantly impact the system’s effectiveness or safety performance.

In summary, while the system has not yet been validated through a physical prototype, its architecture is designed with practical constraints in mind. The outlined mitigation strategies are intended to support a smooth transition from simulation to real-world implementation, and future work will focus on pilot testing and field deployment.

## 6. Conclusions and Future Work

This study investigated the potential of integrating Visible Light Communication (VLC) and Artificial Intelligence to enable adaptive traffic management in complex indoor environments such as airport terminals. Based on simulation results, the performance of a Deep Reinforcement Learning (DRL)-based system was evaluated in terms of multi-intersection coordination and responsiveness to dynamic traffic patterns.

The simulation outcomes suggest improvements in traffic fluidity and pedestrian phase management, as evidenced by metrics such as halting time and message exchange efficiency. VLC proved viable for real-time geolocation and data communication, while the DRL framework, supported by tailored reward functions, demonstrated the ability to mitigate congestion and avoid queuing spillovers in various traffic scenarios.

However, it is important to acknowledge that these conclusions are limited to simulation-based evidence. Aspects such as operational safety, human interaction, and behavior under real-world conditions remain unvalidated. Therefore, broader claims—such as improved safety or human-centric design—should be interpreted as directions for future validation rather than confirmed outcomes.

To move toward real-world applicability, several avenues are envisioned for future research:

*Prototype Development and Pilot Deployment*: As a near-term objective, a functional prototype will be developed incorporating VLC hardware components—specifically multichannel transmitters and amorphous SiC-based receivers—into Automated Guided Vehicles (AGVs) and indoor lighting systems. A controlled pilot deployment is planned in a small section of an airport terminal to assess real-time system performance, signal robustness, environmental adaptability, and interaction patterns.

*Integration with Multimodal Sensing and Communication*: To enhance system resilience, future work will explore the integration of VLC with complementary technologies such as Bluetooth Low Energy (BLE), LiDAR, or Ultra-Wideband (UWB). Sensor fusion strategies will be investigated to ensure positioning accuracy and communication continuity in conditions where VLC performance may be challenged.

*Extended Multi-Agent Learning Scenarios:* The current system employs a single-agent reinforcement learning model. Future research will extend this to Multi-Agent Reinforcement Learning (MARL), enabling cooperative behavior among AGVs and dynamic coordination with pedestrian flows. This includes the development of collaborative traffic strategies and localized priority management under high-density or congested conditions.

By pursuing these directions, the research aims to advance from simulation to real-world deployment, laying the groundwork for scalable, adaptive, and inclusive traffic management systems that are validated in operational environments.

## Figures and Tables

**Table 1:** Communication protocol: synchronization (Sync), type of communication (COM), geolocation (position and time) and payload sections of the transmitted frame. The different colors are assigned to the main components of the protocol.

	SOF 5 bits	Time 6 + 6 + 6 bits			Flag 3 bits	COM 4bits	Position 4 + 4 bits		Payload 4 + 4 + 4 + 4 + 4 + 4 bits						
L2D	Sync	Hour	Min	Sec	END	1	y	x	0000 + 0000						EOF
D2D	Sync	Hour	Min	Sec	END	2	y	x	Lane (0–7)	Device (nr)	Device IDy	Device IDx	Nr. behind	……	EOF
D2I	Sync	Hour	Min	Sec	END	3	y	x	TL (0–15)	Device (nr).	Device IDy	Device IDx	Nr. behind	……	EOF
I2D	Sync	Hour	Min	Sec	END	4	y	x	TL (0–15)	Device ID	Device IDy	Device IDx	Nr. behind	……	EOF
P2I	Sync	Hour	Min	Sec	END	5	y	x	TL (0–15)	N,S,E,W.	……				EOF
I2P	Sync	Hour	Min	Sec	END	6	y	x	TL (0–15)	Phase	……				EOF

**Table 2:** Global percentages of green time for each phase (P 1-9) for Terminals T0, T1, and T2 across standard (pink) and rerouting symmetric (blue) and rerouting asymmetric (green) scenarios. Active phases are labeled at the top for clarity.

** 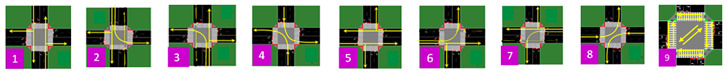 **										
**Standard (% Green Time)**				**Symmetrical (% Green Time)**				**Asymmetrical (% Green Time)**		
	**T0**	**T1**	**T2**		**T0**	**T1**	**T2**		**T0**	**T1**
**P1**	20%	26%	20%	**P1**	16%	14%	25%	**P1**	17%	20%
**P2**	3%	5%	8%	**P2**	2%	5%	3%	**P2**	2%	9%
**P3**	3%	2%	5%	**P3**	3%	2%	5%	**P3**	16%	4%
**P4**	2%	2%	3%	**P4**	5%	5%	2%	**P4**	0%	1%
**P5**	36%	33%	27%	**P5**	41%	26%	19%	**P5**	39%	19%
**P6**	7%	6%	11%	**P6**	5%	17%	11%	**P6**	4%	14%
**P7**	4%	7%	1%	**P7**	7%	8%	13%	**P7**	0%	5%
**P8**	2%	7%	8%	**P8**	1%	3%	3%	**P8**	3%	7%
**P9**	20%	10%	13%	**P9**	16%	17%	16%	**P9**	17%	18%

**Table 3:** Green time allocation (%) per intersection and traffic phase before and after penalization. ↓ decreases, ↑ increases.

Phase	Intersection	Without Penalization (%)	With Penalization (%)	Relative Change (%)
Phase 1 (N–S)	C1	28.0	16.0	↓ 42.9%
Phase 1 (N–S)	C4	27.0	19.0	↓ 29.6%
Phase 1 (N–S)	C0	4.6	4.6	0.0%
Phase 1 (N–S)	C3	4.6	4.6	0.0%
Phase 5 (W–E)	C0	34.0	24.0	↓ 29.4%
Phase 5 (W–E)	C2	20.0	20.0	0.0%
Phase 5 (W–E)	C4	7.0	1.6	↓ 77.1%
Phase 5 (W–E)	C1	16.0	20.0	↑ 25.0%
Phase 6	C1	12.0	13.0	↑ 8.3%
Phase 6	C2	8.0	9.0	↑ 12.5%
Phase 7	C0	17.0	14.0	↓ 17.6%
Phase 7	C1	12.0	13.0	↑ 8.3%
Phase 7	C2	18.0	15.0	↓ 16.7%

## Data Availability

Data is contained within the article.
